# Rodent Models for Investigating the Dysregulation of Immune Responses in Type 1 Diabetes

**DOI:** 10.1155/2013/138412

**Published:** 2013-03-11

**Authors:** Feng-Cheng Chou, Heng-Yi Chen, Shyi-Jou Chen, Mei-Cho Fang, Huey-Kang Sytwu

**Affiliations:** ^1^Department and Graduate Institute of Microbiology and Immunology, National Defense Medical Center, R8324, 161, Section 6, MinChuan East Road, Neihu, Taipei 114, Taiwan; ^2^Department of Pediatrics, Tri-Service General Hospital, 325, Section 2, Chenggong Road, Neihu, Taipei 114, Taiwan; ^3^Laboratory Animal Center, National Defense Medical Center, Taipei 114, Taiwan

## Abstract

Type 1 diabetes (T1D) is an autoimmune disease mediated by T cells that selectively destroy the insulin-producing **β** cells. Previous reports based on epidemiological and animal studies have demonstrated that both genetic factors and environmental parameters can either promote or attenuate the progression of autoimmunity. In recent decades, several inbred rodent strains that spontaneously develop diabetes have been applied to the investigation of the pathogenesis of T1D. Because the genetic manipulation of mice is well developed (transgenic, knockout, and conditional knockout/transgenic), most studies are performed using the nonobese diabetic (NOD) mouse model. This paper will focus on the use of genetically manipulated NOD mice to explore the pathogenesis of T1D and to develop potential therapeutic approaches.

## 1. Introduction 

Type 1 diabetes (T1D) is an autoimmune disease mediated by a combination of genetic and environmental triggers that is characterized by the progressive destruction of insulin-producing cells in the pancreatic islets by autoreactive T cells. It eventually becomes a complex metabolic disease in which patients have insulin insufficiency, dysregulation of blood glucose control, persistent hyperglycemia, and long-term complications. Insulin administration is the most common and widely used therapy to treat T1D. The disease accounts for about 10% of all cases of diabetes, occurs most commonly in people of European descent, and affects approximately two million people in Europe and North America [1]. There is a marked geographic variation in disease incidence, probably because different populations vary in genetic susceptibility/resistance factors (e.g., human leukocyte antigen [HLA] haplotypes) or in exposure to environmental triggers (e.g., Coxsackie virus infection). For instance, a child in Finland (Northern Europe) is about 80 times more likely to develop the disease than a child in China (Eastern Asia) [2]. It is well established that there is a current global increase in the incidence of T1D of 3% per year [3, 4], and this rapid rise strongly suggests that environmental factors are acting on susceptibility genes and contributing to the evolving epidemiology of T1D. 

## 2. Rodent Models for Studying the Pathogenesis of Autoimmune Diabetes

Rats and mice are commonly used animal models for the study of human diseases. Several rat strains and the nonobese diabetic (NOD) mouse spontaneously develop autoimmune diabetes, and these rodent models are commonly used in the study of T1D. They have immunopathological features that resemble the human disease and serve as excellent tools to identify the genetic factors and environmental triggers that lead to the breakdown of immune tolerance. 

### 2.1. Rat Model

The BioBreeding (BB) rat is the oldest, best known, and most extensively studied rat model for the investigation of T1D. This strain is derived from a Canadian colony of outbred Wistar rats in which spontaneous hyperglycemia and ketoacidosis occurred in the 1970s. Affected animals were used to establish all the BB rat substrains, which have distinct immunogenetic backgrounds. The BB/Wor rat is an inbred strain established in Worcester, Massachusetts. The BB rat strain includes both T-lymphopenic diabetes-prone stock and nonlymphopenic diabetes-resistant stock; diabetes can be induced in the latter by manipulating the immune system with transient T-cell depletion combined with either a simulated viral challenge (poly *I : C*) or actual infection with Kilham rat virus or cytomegalovirus (CMV). The BB/Wor rat develops spontaneous and virus-induced syndromes of autoimmune diabetes with high (80–95%) incidence in both genders that serve as good models of human T1D [5]. More recently, two additional inbred rat strains have been established and characterized for T1D development. The first is the Komeda diabetes-prone (KDP) rat, which displays a high incidence of diabetes (approximately 70%) without lymphopenia and 100% development of mild to severe insulitis at 120–220 days of age [6]. The second is the LEW.1AR1 rat, which shows a diabetes incidence of 20% without major sex bias at 58 ± 2 days [7]. These rat models exhibit leukocytic infiltration in the pancreas (insulitis) and develop diabetes spontaneously without sex bias. In addition, these rat strains share a unique major histocompatibility complex (MHC) class II haplotype, *RT*1  *B*/*D* ^*u*^, which may render them susceptible to autoimmune diabetes [8]. 

### 2.2. Mouse Model

The NOD strain was originally developed in Japan during the selection of a cataract-prone strain derived from the outbred Jcl : ICR line of mice [9]. This strain was then established through repetitive brother–sister mating as a subline that spontaneously develops diabetes. The incidence of spontaneous diabetes in the NOD mouse is 60% to 80% in females and 20% to 30% in males. Diabetes onset typically occurs at 12 to 14 weeks of age in female mice and slightly later in male mice. Histological studies have shown that few immune cell infiltrates are noted in islets until approximately 3 to 4 weeks of age, when both male and female mice begin to develop mononuclear infiltrates that surround the islet (peri-insulitis) [10]. The NOD mouse spontaneously develops autoimmune diabetes with immunopathological features resembling those of the human disease, and it can be used as an animal model to study the pathogenesis of T1D.

Multiple loci control the genetic susceptibility to diabetes of this strain. NOD mice harbor a unique MHC haplotype, termed *H*-2 ^*g*7^, which is essential for and is the highest genetic contributor to disease susceptibility [11]. This MHC haplotype does not express an *I*-*E* molecule because of a deletion in the promoter region of *Eα* gene [12]. Moreover, its unique *I*-*A* molecule contains a substitution for aspartic acid at position 57 of the beta chain [13], which substantially alters the repertoire of MHC binding peptides presented by this allele [14].

In summary, the MHC class II molecule in these rodent models determines the susceptibility to T1D, as it does in humans. With regard to immunopathogenesis, it is well established in all the rodent models that T1D is a T-cell-mediated disease, with pathogenic contributions from B lymphocytes primarily as antigen-presenting cells rather than as autoantibody producers.

## 3. Overexpression of Protective Genes in Islets to Escape Immune Cell Attack

In the progression of autoimmune diabetes, *β*-cell damage is mediated by several waves of immune cell infiltration that finally lead to insulin deficiency. The antigen-presenting cells (APCs), such as macrophages and dendritic cells, produce inflammatory mediators (e.g., tumor necrosis factor [TNF]-*α*, interferon [IFN]-*γ*, interleukin [IL]-1*β*, and oxygen free radicals) that initiate insulitis and *β*-cell death [15]. These APCs capture islet antigens and present them to T cells to induce a cell-mediated immune response that selectively destroys *β* cells via the release of cytotoxic molecules (cytokines, granzyme B and perforin) or direct delivery of a death signal via the Fas pathway [16]. Given this knowledge about the mechanisms of *β* cell damage, several islet-specific transgenic mice have been generated in order to dissect the immunopathogenesis of T1D development and to test therapeutic strategies, including the reestablishment of peripheral tolerance, modulation of inflammation, and enhancement of the antiapoptotic activity of the islet.

### 3.1. Regulation of Effector T-Cell Function

It is well established that T cells induce *β*-cell destruction through several proapoptotic pathways, including FasL-Fas interaction, perforin, and TNF-*α*. Previous data have demonstrated that Fas-deficient NOD mice bearing the *lpr* mutation (NOD-*lpr/lpr*) fail to develop diabetes and that irradiated NOD-*lpr/lpr* mice are resistant to the adoptive transfer of diabetes by cells from NOD mice, suggesting that the Fas pathway plays a primary role in *β*-cell death. Consistent with this idea, mice with transgenic overexpression of FasL on *β* cells using the insulin promoter were generated, with the aim of mimicking immunologically privileged sites, so that overexpressed FasL would protect *β* cells from attack by activated T cells that express Fas. Interestingly, these transgenic mice exhibit accelerated diabetes development and display increased sensitivity to diabetogenic T cells. These data suggest that cytokines produced in the islet during insulitis can induce Fas expression on *β* cells, facilitating the transgenic FasL ligation and resulting in a suicide attack [17–19]. To directly attenuate FasL-mediated apoptosis in *β* cells, we generated a decoy receptor 3 (DcR3) transgenic mouse strain and investigated the therapeutic potential of DcR3 in T1D [20]. DcR3 is a soluble receptor that binds to FasL and inhibits FasL-induced apoptosis [21]. Our transgenic mouse data have demonstrated that overexpression of DcR3 in islets results in almost no insulitis and completely inhibits diabetes development without altering the diabetogenic properties of systemic lymphocytes, providing supportive evidence for the crucial role of the FasL-Fas pathway in *β*-cell damage.

T-cell activation requires two important signals: T-cell receptor recognition of a specific peptide-MHC complex and a costimulatory signal. Positive costimulatory signals promote T-cell proliferation and cytokine production, whereas negative costimulation signals induce T-cell anergy [22]. To modulate the activity and properties of infiltrating T cells in the islets, we and others have generated several insulin promoter-driven transgenic mice that overexpress regulatory genes on *β* cells. Programmed death (PD)-1 and cytotoxic T lymphocyte antigen (CTLA)-4 are two important negative costimulation molecules expressed on activated T cells that control their effector functions and tolerance. We have demonstrated that transgenic expression of PD-L1 (ligand of PD-1) [23] or a membrane-bound, agonistic single-chain anti-CTLA-4 Fv antibody (anti-CTLA-4 scFv) [24] on islets in NOD mice reduces the severity of insulitis and suppresses the development of diabetes. However, the role of PD-L1 in the regulation of T-cell tolerance to islets needs to be further investigated because the transgenic expression of PD-L1 on islets in mice with a C57BL/6 background induced T-cell-mediated spontaneous diabetes [25], and transgenic expression of B7-H1 (PD-L1) on peri-islet Schwann cells unexpectedly accelerated rather than suppressed diabetes progression [26]. However, overexpression of positive costimulation molecules (B7.1 and agonistic single-chain anti-4-1BB Fv antibody) on *β* cells disrupts peripheral tolerance and results in the development of intense insulitis and diabetes [27, 28].

### 3.2. Regulation of Cytokine/Chemokine Networks and Overexpression Cytoprotective Molecules

It is well established that proinflammatory cytokines and Th1-related cytokines are highly correlated with disease progression in T1D and are toxic to islets, whereas anti-inflammatory cytokines, such as IL-4, IL-10, and transforming growth factor (TGF)-*β*, are postulated to be protective. To directly address the effects of these protective and destructive cytokines on *β* cells, several islet-specific cytokine transgenic mice were established that allowed dissection of the roles of the cytokines in the islet microenvironment and their subsequent effects on infiltrating lymphocytes. As expected, transgenic expression of IL-4 and TGF-*β* in islets under the control of the insulin or glucagon promoters in NOD mice suppresses insulitis and diabetes [29–31]. However, the *β*-cell-specific expression of TGF-*β* changes the pancreatic architecture [30]. Surprisingly, local production of IL-10 in islets accelerated the onset and increased the prevalence of diabetes [32, 33], suggesting that IL-10 may have diverse functions in addition to its immunoinhibitory effects. It is possible that the timing and location of IL-10 production and the cell types exposed to IL-10 are crucial factors in diabetes development, as systemic administration of IL-10 prevents the onset of diabetes [34, 35]. More recently, a new IL-12 cytokine family member, IL-35, was identified and has been demonstrated to exhibit potent inhibitory effects on effector T cells [36]. Ectopic expression of IL-35 on *β* cells leads to diminished T-cell infiltration and proliferation in the pancreas and long-term protection against diabetes [37]. 

Chemokines are chemoattractants that guide the migration of cells to the inflammatory site. During T1D progression, chemokines that are released by dendritic cells, macrophages, and islets in the inflamed lesion can attract a massive infiltration of leukocytes. We and others have demonstrated that neutralization of chemokines in the islet microenvironment by ectopic expression of decoy chemokine receptors diminishes leukocyte infiltration and prevents diabetes. Overexpression in islets of a pan-chemokine decoy receptor (M3 derived from herpesvirus 68) [38] or an inflammatory CC chemokine decoy receptor (D6) [39] attenuates diabetogenic T-cell accumulation in the pancreas and suppresses the subsequent T-cell activation.

Inflammatory cytokines, such as IL-1*β*, TNF-*α*, and IFN-*γ*, sensitize *β* cells to Fas-dependent and/or other death receptor-mediated apoptosis [40] and induce formation of reactive oxygen species (ROS) in *β* cells. The inhibition of toxic cytokine signaling in islets represents an attractive strategy for designing therapies to prevent islet destruction. Mice with transgenic expression of suppressor of cytokine signaling 1 (SOCS1) in islets showed markedly reduced incidence of diabetes [41]. Disease protection was correlated with the suppression of cytokine-induced signal transducer and activator of transcription (STAT)-1 phosphorylation in SOCS1-expressing *β* cells and with a reduced sensitivity of these cells to destruction by diabetogenic cells *in vivo*. These results suggest that cytokines secreted by effector cells are major contributors to *β*-cell damage. In addition, because islets produce very low levels of antioxidative enzymes and are very sensitive to oxidative stress [42], the reduction of ROS levels in islets is crucial for maintaining the function and viability of islets. We and others have demonstrated that *β*-cell-specific expression of the antiapoptotic and anti-inflammatory proteins thioredoxin (TRX) [43] or heme oxygenase-1 (HO-1) [44] prevents autoimmune diabetes in NOD mice.

Overall, these results of islet-specific expression of immunomodulatory molecules assist in the in-depth dissection of the roles and functions of immune cells recruited to the islets. Most importantly, these results can be further applied to the design of immunotherapies for the treatment of T1D or transplantation rejection [45]. 

## 4. Diabetogenic T-cell Receptor Transgenic Mouse Model to Study Autoimmune Diabetes

T-cell receptor (TCR) transgenic mice have been widely applied in various immunological studies, including investigations of T-cell development, maintenance of peripheral tolerance, control of immune response against infections, and the pathogenesis of autoimmunity. Autoreactive CD4 T cells play a central role in the development of autoimmune diabetes. To study the diabetogenic properties of CD4 T cells, several T-cell clones that respond to the islet antigens presented by *I*-*A* ^*g*7^ were identified and their T-cell receptor repertoires were characterized. This panel of diabetogenic T-cell clones provides valuable information for identifying high-affinity autoantigens and verifying novel autoantigens. These findings can be further applied to develop strategies for the induction of tolerance or to design MHC tetramers for detecting autoreactive T cells [46]. To study the properties of autoantigen-specific T cells *in vivo*, several T-cell receptor transgenic mouse strains have been generated using the TCR *α* and *β* chains from these clones expressed on either CD4 or CD8 T cells.

The BDC2.5 TCR transgenic mice generated using the T-cell receptor gene from the diabetogenic CD4 T-cell clone BDC2.5 are widely used to investigate the T-cell response *in vivo* and *ex vivo* [47]. The BDC2.5 NOD mice develop insulitis within 3 weeks after birth which is much earlier than the wild-type NOD mice, but these mice do not display increased diabetes incidence in either sex. However, when the BDC2.5 TCR transgene is expressed in NOD/SCID mice, the animals develop severe insulitis and diabetes within 4 weeks after birth [48]. Another CD4 TCR transgenic mouse was generated on the NOD background using the TCR genes from the NY4.1 T-cell clone [49]. These NY4.1 TCR transgenic NOD mice develop diabetes in both sexes much earlier than do nontransgenic NOD mice, but the kinetics of disease penetrance in the transgenic and nontransgenic populations is similar. To identify the dominant islet autoantigens that are recognized by diabetogenic T cells, the BDC6.9 TCR transgenic mouse strain was generated to characterize an unidentified antigen that was previously mapped to a locus on chromosome 6 of NOD but not BALB/c mice [50]. The rate of diabetes progression is significantly increased in BDC6.9 TCR transgenic mice; however, when the antigen locus on chromosome 6 of NOD mice was replaced with that from BALB/c mice to generate a BDC6.9 NOD.C6 congenic strain, no diabetes was observed until 1 year of age. More importantly, splenocytes from BDC6.9/NOD.C6 mice retained their diabetogenic properties as demonstrated by an adoptive transfer experiment, which induced diabetes in NOD/SCID recipients with similar kinetics compared to the cells from nontransgenic mice, suggesting that the key islet autoantigen expression is controlled by BALB/c allele on chromosome 6.

Although CD4 T cells are crucial to diabetes progression, mice that lack CD8 T cells develop neither insulitis nor diabetes, suggesting that CD8 T cells participate in *β* cell destruction [16]. To explore the interplay between CD4 and CD8 T cells, TCR genes from the NY8.3 CD8 T-cell clone that recognizes islet-specific glucose-6-phosphatase catalytic subunit-related protein (IGRP) were used to generate an NY8.3 TCR transgenic mouse strain [51]. Similar to the NY4.1 TCR transgenic mice, NY8.3 TCR transgenic mice show an accelerated onset of diabetes compared with nontransgenic NOD mice. Interestingly, when the NY8.3 TCR transgene is crossed to a RAG2-deficient NOD background, the mice, which have only NY8.3 CD8 T cells and no CD4 T cells, develop diabetes less frequently and significantly later than do RAG-2-sufficient NY8.3 TCR transgene NOD mice. These results emphasize the notion that CD4 T cells play a key role in diabetes development.

Because of the heterogeneity of the islet-reactive CD4 and CD8 T cells in NOD mice, using the TCR transgenic mice that carry a monoclonal TCR *in vivo* may simplify the experimental system for testing specific immune reactions. In addition, T cells from TCR transgenic mice provide a source of naïve T cells for the *in vitro* generation of self-antigen-specific Th1, Th2, Th17, and regulatory T cells, whose pathogenic and protective roles can then be unambiguously examined [52–54]. Thus, the TCR transgenic mice provide *in vivo* models to explore the polymorphism of endogenous autoantigens, to determine the crucial autoantigens at the initiation of disease development, and to identify key epitopes of the autoantigens. In summary, established and well-characterized TCR transgenic mouse lines are available to assist researchers in understanding the pathogenesis of autoimmune diseases and developing therapeutic strategies, for example, mapping autoantigens recognized by T cells and evaluating autoantigen-specific tolerance therapy.

## 5. Models of Virus-Induced Disease and Transgenic Expression of Islet-Specific Neoantigens in the Study of Peripheral Tolerance

T1D has been associated with viral infections including enteroviruses, rubella, mumps, rotavirus, parvovirus, and cytomegalovirus. Of these, Coxsackie virus is the most common enterovirus found in prediabetic and diabetic individuals. This virus can infect a number of tissues and primarily causes severe pancreatitis that may lead to the induction of autoimmune diabetes through molecular mimicry [55] or bystander activation of autoreactive T cells [56]. 

By contrast, accumulating evidence from epidemiological observations and experimental animal data suggests that viral infections can prevent T1D [57]. Thus, the data concerning the effects of viral infections on either enhancement or prevention of T1D are not conclusive, and many factors must be considered. For example, the replication level of the virus can be important; enteroviruses that replicate at higher levels accelerate the development of T1D, whereas lower replication levels result in the prevention of diabetes [58]. Moreover, host genetic factors also influence the outcome of viral infection. Genome-wide association studies (GWAS) and target gene sequence analysis have shown that genetic variations mediate differential host cell responses to viral infections that eventually promote or prevent T1D [59].

Autoreactive T cells in the body are elegantly controlled by negative selection through the induction of apoptosis in the thymus [60], and those self-reactive T cells that escape to the periphery are suppressed by regulatory T cells [61]. To study the mechanisms that maintain immunologic self-tolerance in the periphery, several strains of transgenic mice with islet-specific expression of exogenous proteins (using the insulin promoter) have been established to study the T-cell response *in vivo* [62–65]. In these models, the expression of viral proteins (glycoprotein from lymphocytic choriomeningitis virus or hemagglutinin from influenza virus) on *β* cells did not induce T-cell-mediated destruction under steady-state conditions, demonstrating T-cell ignorance in the periphery. However, infection of these transgenic mice with viruses that carry these antigens abolished peripheral tolerance, resulting in T-cell-mediated diabetes. Importantly, these data also indicated that breaking tolerance is dependent on the maturation and activation status of APCs. Viral components recognized by host pattern recognition receptors (PRRs) expressed on APCs can induce the functional maturation of APCs and the presentation of antigens to T cells, resulting in the activation of T-cell responses [66].

In summary, these mouse models have been instrumental in increasing our knowledge of the relationships among environmental infections, genetic variation, and host immune responses. Moreover, these models can be further applied to investigate the molecular mechanisms of clonal deletion and clonal anergy and to understand the balance between the activation of effector cells and immune tolerance or ignorance.

## 6. Humanized MHC Transgenic Mice in the Study of T-Cell Autoreactivity

NOD mice also serve as a model for identifying the genetic factors that predispose a host to the immune dysregulation involved in development of autoimmune diabetes [67]. Among those susceptibility loci, the MHC molecules within the *Idd1* locus confer the major proportion of disease susceptibility. Genetic studies searching for diabetes susceptibility genes have identified more than 60 loci that contribute to susceptibility to T1D in humans. The products of these loci have been extensively investigated in order to understand their molecular mechanisms and to develop genetic prediction methods that show promise for use in preventive strategies [68]. Interestingly, more than 90% of patients who develop clinical diabetes have particular MHC haplotypes, which is also the case in autoimmune-prone rodent models [69]. NOD mice have a unique *I*-*A* ^*g*7^ haplotype that expresses an uncharged serine residue at position 57 of the *A*_*β*_ chain, in contrast to other diabetes-resistant strains that use a negatively charged aspartic acid, suggesting that this change leads to diabetic susceptibility [13]. The homolog of mouse *I*-*A* ^*g*7^ in humans is the HLA-DQ8 (*DQA*1*0301/*DQB*1*0302) MHC class II molecule, which also encodes an uncharged serine, alanine, or valine residue at *β*57. Given this genetic nature of disease susceptibility, several groups have used humanized MHC transgenic models to understand the molecular mechanisms of the specific TCR/peptide/class II interactions involved in the disease process and to map T-cell epitopes for a variety of human islet autoantigens presented by MHC class II molecules, for example, the DQ8 molecule [70]. 

In 1999, two independent groups generated a DQ8 transgenic mouse in an *I*-*Aβ* mutated NOD mouse strain with the nature of null *I-E* allele that prevents the expression of endogenous MHC class II molecules. In these mice, the majority of CD4^+^ T cells are restricted to the DQ8 molecule and can be used to identify dominant T-cell epitopes, such as glutamic acid decarboxylase 65 (GAD65) [71, 72]. These mouse models are useful tools to define the important epitopes of autoantigens that are processed by antigen-presenting cells and recognized by human T cells. Furthermore, these analyses provide an important resource for investigating diabetes pathogenesis and for developing antigen-specific therapies and strategies for T-cell monitoring during disease development and therapeutic intervention [73]. Although the NOD human CD4 transgenic/DQ8 transgenic/*I*-*A* ^null^ mice were found not to develop autoimmune diabetes [72], subsequent studies have addressed in detail the issues regarding disease-resistant and susceptible HLA-DQ alleles *in vivo* by crossing these mice with *β*-cell-specific B7-1 transgenic mice [74]. Wen et al. generated DQ8 transgenic (DQ8tg) mice in an MHC class II-deficient (mII^−/−^) C57BL/6 strain that is free from the potential influence of the other diabetes susceptibility genes in the NOD strain. However, similar to the earlier transgenic mice generated on the NOD background [72], these humanized mice did not spontaneously develop diabetes. Interestingly, when the DQ8tg/mII^−/−^mice were mated with a strain expressing a rat insulin promoter-driven costimulation molecule B7.1 transgene, around 81% of these DQ8tg/mII^−/−^/RIP.B7tg  mice spontaneously developed diabetes. Strikingly, replacement of DQ8 with a diabetic resistant DQ6 molecule in the same mice set to generate DQ6tg/mII^−/−^/RIP.B7tg mice, which developed neither insulitis nor diabetes [74]. 

In humans with T1D, the most common HLA haplotype contains the DR4 and DQ8 molecules, and the two disease-associated molecules are in strong linkage disequilibrium. To further dissect the relative importance of the roles of DQ8 and DR4 in diabetes development *in vivo*, Wen et al. established DR4tg/mII^−/−^/RIP.B7tg and DQ8DR4tg/mII^−/−^/RIP.B7tg mice that monitored the pathogenesis of T1D in these mice [75]. Surprisingly, only 25% of DR4tg/mII^−/−^/RIP.B7tg mice developed diabetes, and the simultaneous expression of DR4 and DQ8 molecules in the mII^−/−^/RIP.B7tg mice resulted in reduced diabetes incidence (23%) compared with the DQ8tg/mII^−/−^/RIP.B7tg mice (81%). The authors suggested that the DR4 molecule downregulates the diabetogenic effect of DQ8 by enhancing Th2-like immune responses.

Thus, these humanized models can be applied to identify crucial epitopes of the autoantigens that are restricted by disease-susceptible MHC molecules. More importantly, the epitopes identified in these systems are naturally processed and presented by APCs, which support the possible clinical relevance of these epitopes. These findings could ultimately be exploited to monitor autoimmune activity in at-risk individuals or patients undergoing intervention therapies [73]. 

## 7. Conclusion

In summary, using animal models to study the pathogenesis of T1D circumvents the ethical and technical problems that cannot easily be resolved in humans. The benefits of using animal models include (1) ease of access to pancreata and pancreatic lymph node, which can allow direct analysis of the status of the lymphocytes in the inflamed lesion; (2) evaluation of potential therapeutic strategies and determination of the dose-dependent effects and optimal timing of treatment interventions under stringent controls (e.g., housing animals in specific pathogen-free conditions); (3) simplicity of identification of the susceptibility/resistance loci in the inbred rat/mouse. However, some trials of immunomodulatory therapy that have succeeded in the NOD mouse model have failed in human clinical trial [76]. Therefore, the findings in the animal models should be further considered before translation into the clinic. In the next generation of studies, GWAS may be used to map genomic regions other than the MHC genes that contribute to the susceptibility of humans to T1D [77], which could lead to the creation of more genetically modified NOD mouse models to explore T1D genotype-phenotype relationships.
